# Liquid marbles, floating droplets: preparations, properties, operations and applications

**DOI:** 10.1039/d2ra00735e

**Published:** 2022-05-19

**Authors:** Yukai Sun, Yelong Zheng, Chuntian Liu, Yihan Zhang, Shiying Wen, Le Song, Meirong Zhao

**Affiliations:** State Key Laboratory of Precision Measuring Technology and Instruments, Tianjin University Tianjin China zhengyelongby@tju.edu.cn

## Abstract

Liquid marbles (LMs) are non-wettable droplets formed with a coating of hydrophobic particles. They can move easily across either solid or liquid surfaces since the hydrophobic particles protect the internal liquid from contacting the substrate. In recent years, mainly due to their simple preparation, abundant materials, non-wetting/non-adhesive properties, elasticities and stabilities, LMs have been applied in many fields such as microfluidics, sensors and biological incubators. In this review, the recent advances in the preparation, physical properties and applications of liquid marbles, especially operations and floating abilities, are summarized. Moreover, the challenges to achieve uniformity, slow volatilization and stronger stability are pointed out. Various applications generated by LMs’ structural characteristics are also expected.

## Abstract 2

The recent advances in the preparation, physical properties and applications of liquid marbles, especially operations and floating abilities, are summarized.

## Graphical abstract

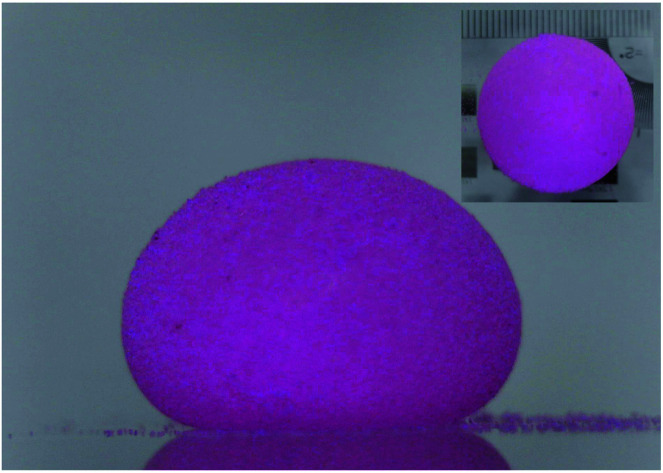

## 1 Introduction

**Fig. 1:**
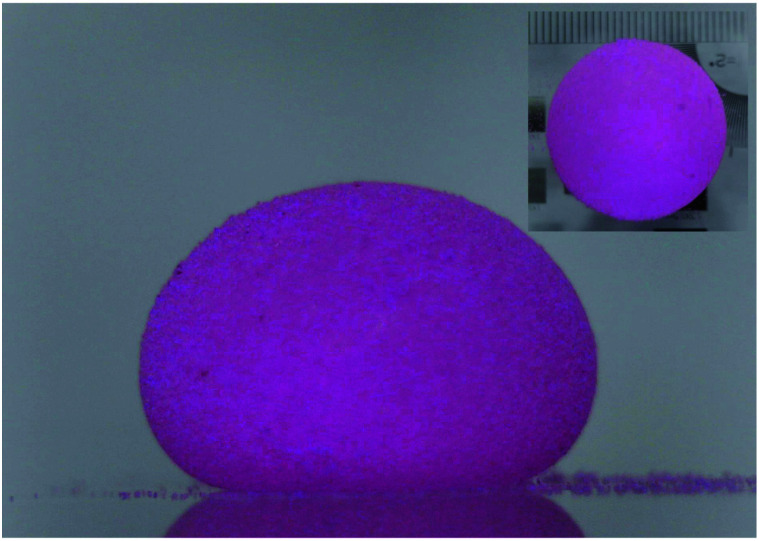
A typical 35 μL LM packaged with lycopodium.

LMs (Fig. 1), referring to non-wettable spherical droplets coated with a layer of nanoparticles, were firstly reported by Aussillous and Quéré in 2001.^1^ Traditionally, LMs can be formed by rolling droplets on the nanoparticle layer. The coating shell could prevent the liquid from wetting the outside surface.^2,3^ Moreover, the coating shell allows the LM to float on a liquid surface.^4^ Compared with the rolling method to prepare LMs,^5–10^ more efficient preparation methods have been proposed.^11–19^ Particles such as polyvinylidene fluoride (PVDF),^11,12,20–23^ graphite^24–26^ and carbon black^5,27^ are commonly used for LMs.

Due to the special characteristics, LMs play significant roles in various fields. For example, liquids in LMs, such as water or blood, are more operable.^28^ LMs can be driven or disassociated by external stimuli^29,30^ and provide a cozier environment for an aerobe.^2,31^ The particle shell ensures longer-time stability^24,32–35^ and stronger robustness of LMs because of the decreased evaporation rate.^32,36–43^

LMs have a broad application prospect in microreactors and biological incubators. The marbles provide a 3D microenvironment for microchemical reactions^26,28,44–47^ and cell culture.^2,3,19,26,31,48–56^ They can keep intact even if falling from a certain height or under certain pressure. The smaller volume can reduce the demand for the reaction solution and speed up the heat and mass transfer processes.^2,3,57,58^ The controlled on–off motion allows the inner reactant to be added or extracted smoothly without being ruptured.^59–62^ LMs can also transport the required liquid and achieve directional movement by external stimuli, such as magnetic force or light. So LMs can be utilized as vehicles for targeted drug delivery.

In this review, the significant progress in the preparation, properties and applications of LMs is presented. Finally, the challenges of LMs and their application prospects are also put forward.

## 2 Liquid marbles preparations

The wide sources of raw materials and easy preparation methods lay a solid foundation for the vigorous development of liquid marbles. In this section, we have summarized many researchers’ preparation methods.

### 2.1 Materials

LMs can be produced from a wide range of materials. According to the required application, different types of liquids and particles are used. Usually, the encapsulated liquid refers to water,^1^ aqueous surfactant solution,^25^ organic solvents,^60^ ionic liquid,^63^ molten metal,^64–66^ reaction reagents,^44,60,67,68^ blood,^69^ cell culture mediums,^49^ or even viscous adhesive.^70^ In previous studies, the coating particles are mainly lycopodium,^1,12,23,71–74^ Fe_3_O_4_ nanoparticles,^60,61^ graphite,^24–26^ carbon black,^5,27^ copper powders^6,18^ or polytetrafluoroethylene (PTFE).^23,35,75–79^ The particles used in LMs from previous literature reports are presented in Table 1.

**Table 1:** Powders used to prepare liquid marbles

Powder	Hydrophobicity	References
Lycopodium	Hydrophobic	1, 12, 14, 23, 27, 31, 71–74 and 80–82
PTFE	Hydrophobic	12, 14, 23, 31, 35, 48, 57, 63, 66, 74–79 and 83–88
Polyethylene (PE)	Hydrophobic	12, 14, 23, 80 and 81
Silica powder	Hydrophobic	1, 10, 16, 71, 75 and 89–98
Hydrophobized copper	Hydrophobic	6 and 18
Fe_3_O_4_ nanoparticles	Hydrophobic	60, 61, 99 and 100
PVDF	Hydrophilic	11, 12, 14, 20–23, 74, 80, 81, 88 and 100
Graphite	Hydrophilic	24–26
Carbon black	Hydrophilic	5, 14 and 27
Janus particles	Hydrophobic/hydrophilic	98

### 2.2 Methods of producing LMs

Preparing stable liquid marbles is fundamental to the manipulation and application. The common method to prepare LMs with composite functions is rolling droplets on a powder bed.^5,13,50,92,104,105^ Except for the rolling approach, many researchers have proposed other ways to prepare LMs (see Table 2), such as electrostatic formation,^30,79,95,102,106–109^ self-organization by evaporation,^96,97^ impacting hydrophobic powders,^77,103,110,111^ self-assembly on a water surface,^16,18,50,98^ inclined rolling method,^15,43,101,112^ rotary centrifugation,^19^*etc.*

**Table 2:** Methods of producing LMs

Methods	Advantages	Disadvantages	References
Rolling	Easiest	Inconsistent coverage	5, 13, 50, 92, 104 and 105
Electrostatic formation	Customizable	Time and energy consuming; material requirements	30, 79, 95, 102 and 106–109
Self-organization by evaporation	Spontaneous	Time consuming	96 and 97
Self-assembled on the water surface	Multifunctional	Time consuming and inaccurate volume	16, 18, 50 and 98
Impacting hydrophobic powder	Batch; simplify the preparation process	Droplet sputtering; inaccurate volume	77, 103, 110 and 113
Inclined rolling method	Batch	Droplet sputtering, inaccurate volume	15, 43, 101, 112 and 114
Rotary centrifugation	Multifunctional; volume accuracy	Uncontrollable shape and inconsistent coverage	19

#### 2.2.1 Rolling method

The easiest method to prepare LMs is as follows: a droplet is injected into a layer of hydrophobic powder, and then the droplet is rolled over the surface of the powder (the powder would automatically attach to the droplet). An LM can be formed until the droplet is completely covered by the powder.

**Fig. 2:**
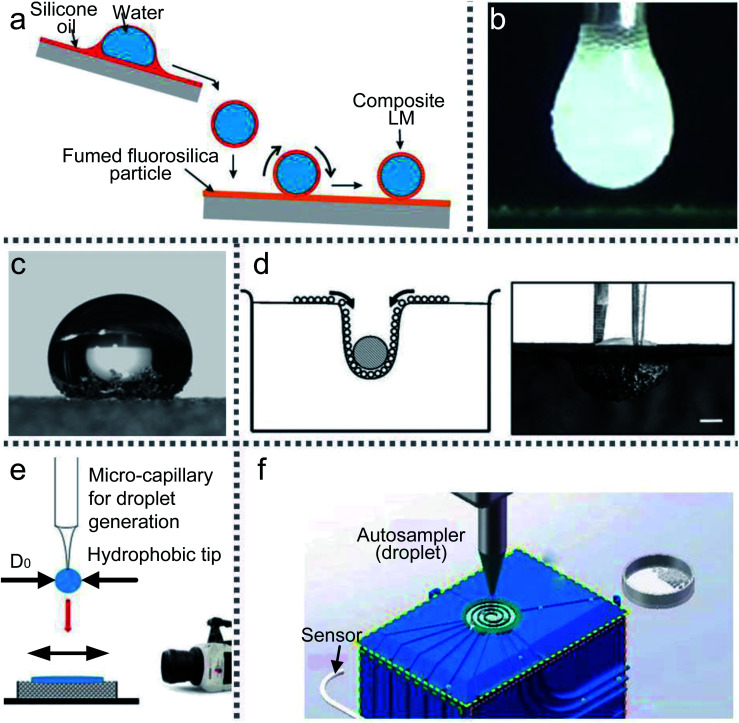
Various preparation methods for LMs. (a) Inclined rolling method. Reproduced from ref. 101 copyright 2020, Elsevier. (b) Electrostatic formation of liquid marbles. Reproduced from ref. 102 copyright 2016, Elsevier. (c) Self-organization by evaporation. Reproduced from ref. 96 copyright 2007, AIP Publishing. (d) Self-assembled on the water surface. Reproduced from ref. 18 copyright 2008, John Wiley and Sons. (e) Impacting hydrophobic powders. Reproduced from ref. 103 copyright 2016, Elsevier. (f) Rotary centrifugation. Reproduced from ref. 19 copyright 2019, John Wiley and Sons.

Moreover, when a droplet is directly released on a ramp which is coated with superhydrophobic materials, the ramp would accelerate the slide and form a liquid marble.^15,114^ If part of the ramp surface is covered with two different powders, this method (inclined rolling method) can be used to generate composite LMs. Adjusting the liquid type and droplet volume can fabricate the required LMs. Thus the inclined rolling method (Fig. 2(a)) has great potential for the emerging digital microfluidic platform, biomarker and micro-reactor/bioreactor.^101^

#### 2.2.2 Electrostatic formation

A conductive substrate covered by micron-sized particles gradually moves upwards to approach a pendent water drop.^115^ Owing to the electrostatic interaction, the powder would automatically adhere to the droplet. Water-based LMs of complex multilayered morphologies can be formed by hydrophilic or hydrophobic particles.^116^ However, this method is time-consuming and energy-intensive compared with the traditional rolling method (especially for particles with weak electrical conductivity). The particle conductivity is crucial in electrostatic aggregation.^108^ Increasing the conductivity of the particle shell could reduce particle movement in the same field strength. In 2013, Liyanaarachchi *et al.* published a paper, in which they first described the preparation of LMs by the electrostatic method.^79^ In 2016, Ireland *et al.* and Jarrett *et al.* produced LMs by an electrostatic field and respectively analyzed the effect of the droplet and particle size on the LMs’ stability (Fig. 2(b)).^95,102^ They found that using smaller droplets and larger particles can easily produce stable liquid marbles.

Likewise, Jarrett prepared LMs using silica, coal and sphalerite particles by electrostatic fields. The particles’ wettability, conductivity, shape, size and density all together determine the structures’ shape and internal composition of the LMs. Recently, Kawata used PNIPAM@PS powder to form LMs *via* static electricity and the voltage power could reach 2.0 kV.^30^ Kido prepared LMs with pH-responsive particles in two ways: rolling and the electrostatic method. They demonstrated that the powders from pH 3.0 solution are hydrophilic but from pH 10.0 solution are hydrophobic.^104^

#### 2.2.3 Self-organization by evaporation

The coating process can be driven by self-evaporation, which takes inspiration from soil’s waterproof property after forest fires or oil spills (Fig. 2(c)).^96^ Under the effect of self-evaporation, the mixed hydrophobic–hydrophilic particles can aggregate into a hydrophobic shell which can wrap the liquid core. Initially, when the droplet is placed on the powder surface, a few particles would attach to the bottom of the droplet. But over time these particles can climb higher until the droplet is covered by a complete coating and appears as a liquid marble.

Bhosale (2012) reported a novel evaporation/condensation method to continuously produce liquid marbles with the average diameter of particles ranging from 3 to 1000 μm.^97^ The primary mechanism causing the formation of liquid marbles is droplet nucleation by condensation. Drop coalescence is the second mechanism to destroy the distribution width controllability. A thin layer of hydrophobic powder is spread on the liquid-free surface in a glass vial. The glass vial is placed on a heating pad and heated from the bottom. Then the liquid-vapor released at the free surface is condensed and enveloped by the particulate material at the surface. In the end, stable liquid marbles are produced and recovered from the sweating process.

#### 2.2.4 Self-assembled on the water surface

Floating a droplet on granular rafts can also fabricate LMs. A self-assembled particle sheet can support objects to sink into the water slowly (Fig. 2(d)).^18^ Injecting a droplet on the particle sheet can cause a particle meniscus on the water surface. As the droplet volume increased, the LMs would sink into the liquid bottom and form a complete liquid marble. Kim (2010) investigated superhydrophobic microspheres inspired by superhydrophobic insects. Dropping water onto the monolayer microspheres can form a spherical droplet with a diameter of 2.4 mm, whose surface is coated with superhydrophobic hemispheres.^98^ Compared with the height and aspect ratio of floating a LM on the water surface, the theoretical model of the rigid sphere can also be applied to LMs.^117^

SiO_2_ particles can prevent droplets from coalescing into the oil–water interface.^16^ A droplet is placed at the center of a SiO_2_ particle raft using a syringe. Whether the droplet can achieve its maximum volume mainly depends on the particle size and density. The volume of the floating droplet is gradually increased until the raft is unstable. Then the droplet would sink into the liquid bottom and be covered with the oil–particle film. The floating droplet’s shape, the raft deformation and the complex capsule shell can be predicted by modeling the raft.

#### 2.2.5 Impacting hydrophobic powders

Kinetic energy can enlarge the contact area between the liquid and powder during the LM’s impacting and rebounding, which ensures the particles adhere to the liquid surface. The increase in kinetic energy and the decline of particle size can promote the percentage of particle coverage. Eshtiaghi calculated the solid–liquid spreading coefficient (*λ*_SL_) and examined the impacting of LMs using various liquids and particles.^77^ They showed that both kinetic energy and particle size determine the coverage of the LMs while spreading coefficients do not affect it.

The final shape of droplets falling onto powders is different from that of solid spheres.^110^ When the impacting speed is above the critical value, the droplet would deform to a non-spherical shape. But it has a near-complete particle coverage on the LM’s surface, which could freeze oscillations. In 2016, Supakar also experimented with the impacting of droplets onto powders (Fig. 2(e)). They assessed the critical conditions for forming LMs by varying the volume of the droplets, the impacting speed and the size of powders.^103^

There are still challenges in the preparation processes, especially continuous preparation.^111^ The impacting of droplets on the particle bed presents a simple and fast approach to fabricate LMs, which could expand the scope of applications in microfluidics. Taken together, these studies support that the quality of LMs is determined by the kinetic energy, hydrophobicity of the powders and the size of droplets. Compared with the rolling method, the impacting method simplifies the preparation work for LMs. Unfortunately, liquid sputtering may occur during the rebounding, which would lead to an inaccurate volume for the LMs.

#### 2.2.6 Rotary centrifugation

Rotary centrifugation can manufacture controllable and reproducible LMs. An automated system uses the centrifugal force and gravity (Fig. 2(f)) to achieve rapid formation, coalescence and splitting of LMs. The size of the LMs generated from the system would be extended from micrometer to sub-millimeter scales. The highly automated process could ensure the uniformity of LMs.^19^

Since the armored capsules’ content is isolated, transportable and easily releasable, the LMs are great candidates for applications such as green chemistry and cell biology. The preparation of LMs is the basis of manipulations and applications. Certain materials and methods to produce LMs are reviewed above (Table 2), among which the rolling method is recognized as the easiest and cheapest way.

## 3 The properties of LMs

### 3.1 Floating abilities

**Fig. 3:**
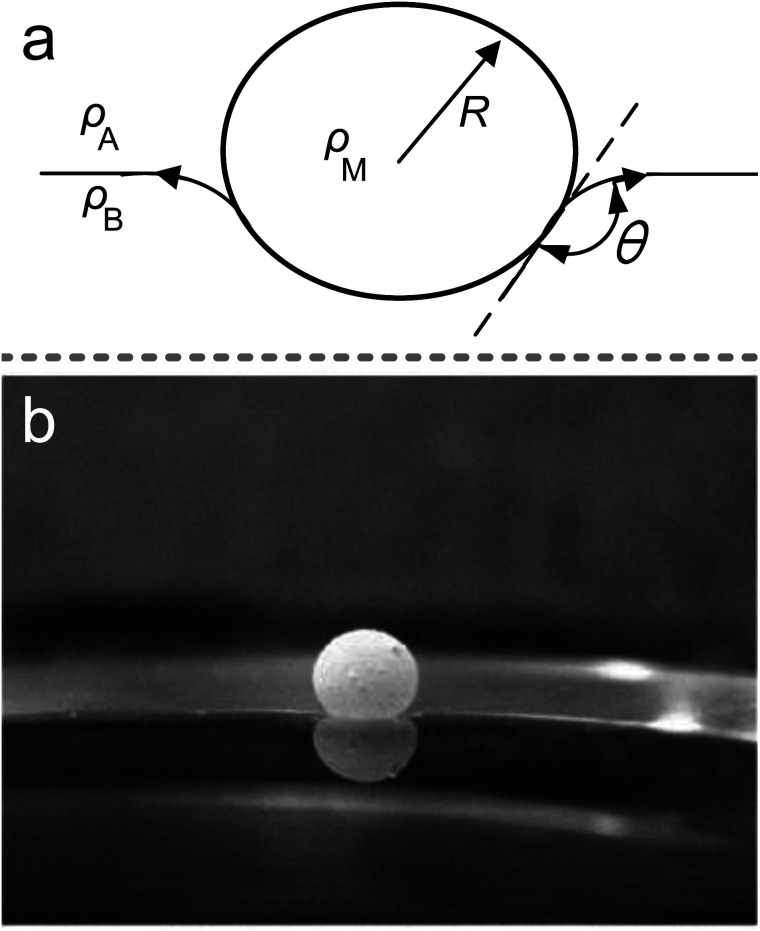
The marble supported by a liquid surface. (a) Diagram of the floating marble. (b) A 10 μL marble floating on NaCl solution. Reproduced from ref. 21 copyright 2009, Elsevier.

It is well-known that the surface tension of the liquid–air interface allows small objects to float on the liquid surface, even if their density is substantially larger than that of the carrier liquid (Fig. 3(a)); *e.g.*, water-walking creatures avoid drowning by surface tension. The LMs can also float on the liquid surface due to a layer of air between the liquid marble’s shell and the carrier liquid.^118^ The air gap could prevent the LMs from directly contacting with the carrier liquid and provide a relatively large contact angle, which is similar to the Cassie–Baxter state. When the LMs float on the liquid surface, both the marble and the liquid surface are deformed (Fig. 3(b)).^21^ The force balance equation is *F*_w_ = *F*_s_ + *F*_b_ for a floating body from the generalized Archimedes’ principle (*F*_w_ is the weight of floating body, *F*_s_ is the surface tension force and *F*_b_ is the buoyancy force).^21,117^ If spreading hydrophobic powders on the liquid/air interface and depositing marbles on the interface, the contact angle would be close to 180°.

LMs on liquid would extend the marble’s lifetime since the high humidity can reduce the evaporation rate. This is the main reason why researchers pay particular attention to the floating abilities of LMs.

### 3.2 The elasticity of LMs

**Fig. 4:**
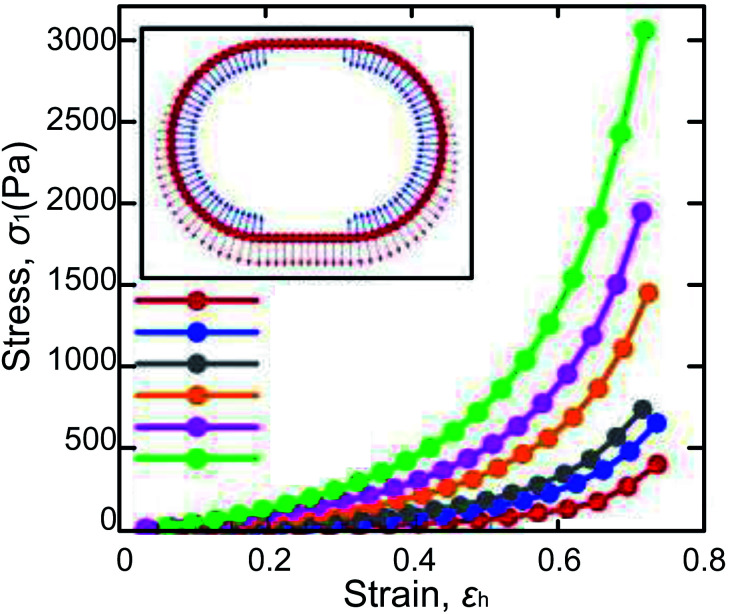
Variations of *σ*_1_*vs. ε*_h_ obtained from the numerical model of LMs, whose inner liquids have different surface tensions. Reproduced from ref. 37 copyright 2019, AIP Publishing.

Surface tension and capillary force on the shell ensure the LMs’ excellent elasticity, which can resist compression and impact. To assess the elasticity of LMs, an LM is compressed between two parallel hydrophobic glass slides until the LM is ruptured at a relative compression rate of 53%.^32,36^ The compression of LMs is a quasi-elastic process and the pressure of the slide is mainly converted into surface energy.^38^ Moreover, the LMs’ model can evaluate the effect of the volume, density, and surface tension on the stress–strain coefficient. Polwaththe-Gallage (2019) squeezed an LM on an electronic balance with a linear stage. Eventually, the LM can be deformed vertically by approximately 0.7*h*_0_ (*h*_0_ being the original height of marble, Fig. 4).^37^ Liu (2019) redefined the description of rupture dynamics for LMs.^43^ And a mathematical model is depicted to predict the theoretical density of particles at rupture. The result suggested that the gradient of particle density from center to edge may lead to rupture and the cracks always appear at the edge of the contact area between the marble and the substrate. Azizian (2019) utilized two different hydrophobic CaCO_3_ powders to make LMs which show different mechanical responses under compression.^39^ It indicates that the difference in the elasticity of LMs arises from the arrangement of particles. Furthermore, to investigate the plasticity of LMs, the LM is squeezed several times. The result shows that the relative compression rate could reach 65% and the height of the recovered LM decreases at 5%. The main factors affecting compression behavior are the structure of shells and compression/decompression rate.

Two questions about the elasticity study of LMs are raised:^40^ (1) although the force to compress LMs depends on their volume, it is unclear whether the particle shell plays a role. (2) Particle coating determines whether the marble can be compressed without being ruptured. To validate the fact that the elasticity of LMs is independent of the shell, a truncated oblate spheroid model is built to calculate the surface area during LM’s deformation. The curves show that the trend of the compression force is consistent with that of the surface area and indicates that the rupture of marbles is determined by the increment of surface area.

Here’s another different experiment performed by Asher.^41^ An LM on a mass balance is gradually compressed using a plastic head and the balance data is recorded. The elasticity of the LMs is mainly provided by the liquid menisci between particles. Liu (2015) investigated the mechanical robustness of LMs by changing the size of particles and the type of liquid.^42^ He found that the squeezed liquid marbles would rupture when the particles on the air–water interface become sparse.

Much research has investigated the elasticity or mechanical robustness of LMs. Since elasticity or mechanical robustness represents the ability of LMs to resist high pressure, shocks, impacting, deformation, *etc*., it is particularly crucial to the transportation and manipulation of LMs.

### 3.3 Reductions of evaporation rates

Evaporation and rupture would destroy the initial shape of LMs and affect their applications. Since the surface of the LMs is covered by hydrophobic particles and the interfacial area between internal liquid and air decreases, the evaporation of liquid inside a LM is usually slower than that of the exposed droplet.^32^ If LMs could be given a longer lifetime, they could have more time to transport the inner liquid to the destination.

The shell structure of LMs can slow down the evaporation rate, mainly due to the number of layers, the diameter of hydrophobic particles and the chemical nature of hydrophobic particles, *etc*. Laborie reported an evaporation experiment, in which the water surface was coated with a single or several layers of hydrophobic particles.^33^ He showed the relationship between the liquid–air interfaces and evaporation rate.

To analyze the factors affecting the evaporation rate of LMs, four different powders: polytetrafluoroethylene (PTFE), ultrahigh density polyethylene (PE), Ni and a mixture of Ni with PE (Ni–PE) were used to prepare LMs and study the evaporation under the same ambient conditions.^34^ The results show that the PE LMs have the lowest evaporation rate. In 2013, Cengiz also investigated the factors affecting the evaporation rate of LMs. The marbles were formed by polytetrafluoroethylene powder (7 μm) and ultra-hydrophobic poly(perfluoroalkyl ethyl acrylate) powders with three different particle sizes (8, 20, and 60 μm).^87^ They found that particle size, surface energy and hydrophobicity of the powder co-determine the evaporation rates of the LMs.

**Fig. 5:**
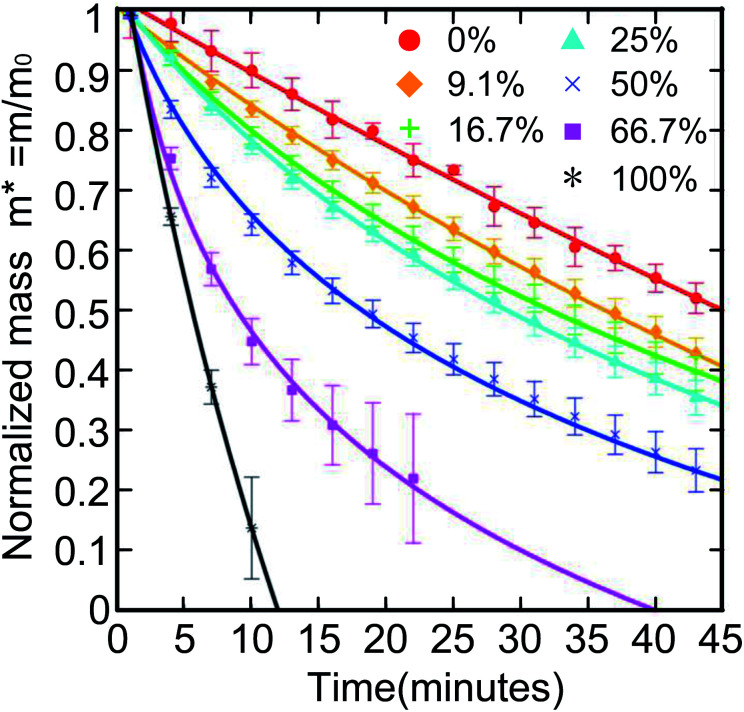
Normalized mass of a 10 μL aqueous ethanol LM with different ethanol volume concentrations. The LMs with higher ethanol concentration have a steeper curve. Reproduced from ref. 119 copyright 2016, American Chemical Society.

The environment can also affect the evaporation rate of LMs, such as temperature or relative humidity (RH). Saturated solution can maintain constant relative humidity and keep the cell between 20 and 26 °C.^24^ When the temperature and RH reach an equilibrium state, the evaporation rate of LMs is monitored. The evaporation rate would decline as the RH declined. If the liquid inside the LM is mixed with ethanol, its service life will also decay (Fig. 5).^119^ Sreejith first prepared liquid marbles for polymerase chain reaction (PCR), using a humidity-controlled chamber to reduce the evaporation rate.^122^

### 3.4 Coalescence of LMs

LMs have excellent elasticity and stability, which can avoid the merging of two LMs under proper extrusion and collision conditions. The powders of LMs prevent the formation of liquid bridges.^19^ The elasticity of LMs enables them to sustain the repeatable deformation up to 53%.^32,36^

**Fig. 6:**
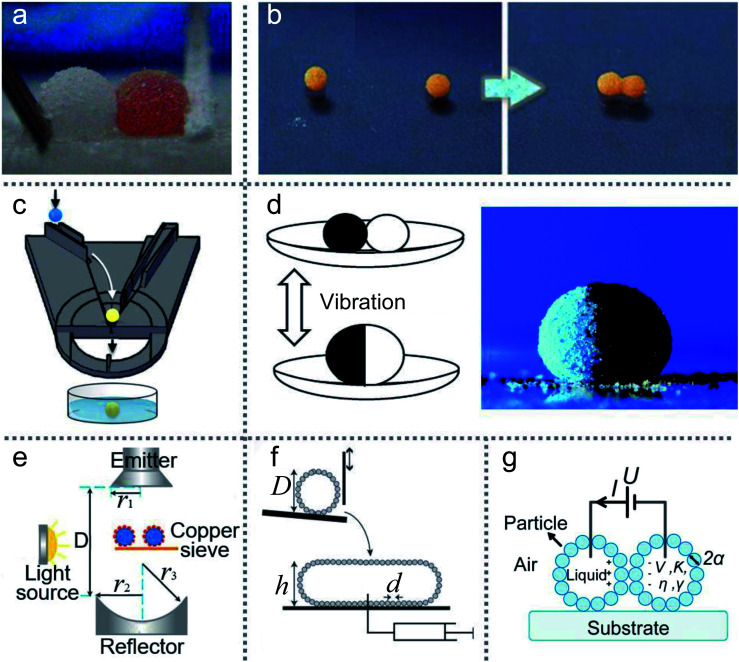
Methods of merging liquid marbles. (a) Mechanical extrusion. Reproduced from ref. 118 copyright 2017, American Chemical Society. (b) Coalescence induced by magnetic fields on the water surface. Reproduced from ref. 120 copyright 2013, American Chemical Society. (c) Merging by a 3D printed slide platform. Reproduced from ref. 15 copyright 2016, American Chemical Society. (d) Preparation of composite liquid marbles by vibrations. Reproduced from ref. 27 copyright 2011, American Chemical Society. (e) Coalescence by ultrasonics. Reproduced from ref. 45 copyright 2017, American Chemical Society. (f) Coalescence by impacting. Reproduced from ref. 121 copyright 2013, AIP Publishing. (g) Liquid marbles’ coalescence by electric fields.

To merge two LMs, external conditions need to be provided, such as magnetic fields or DC voltages. The simplest way to merge LMs is using tools to squeeze two LMs directly. Sivan formed two marbles by rolling HCl-treated Galinstan droplets on an 80 nm WO_3_ powder bed, and then mechanically pressed them together.^66^ Similarly, Bormashenko directly pressed two LMs using a plasma hydrophilized glass rod (Fig. 6(a)) resulting in the forced coalescence.^118^

A LM with a magnetic coating or magnetic solution can be accurately positioned and moved on solid or liquid surfaces *via* a magnetic field.^62^ LMs coated with composite powders of FD-POSS and Fe_3_O_4_ can be actuated to move in different directions or open/close repeatedly under the magnetic field.^60^ Droplets coated with hydrophobic Fe_3_O_4_ nanoparticles are enabled to open and close repeatedly under the magnetic field.^61^ Moreover, the magnetic force could open the coating of magnetic LMs to initiate coalescence. The coalescence of LMs would become a spontaneous process when liquid-to-liquid contact occurs, since the total surface area and surface energy of LMs tend to reduce. The two liquid marbles can be opened from the top to bottom under the force of the magnetic field, and then both the exposed droplets coalesce immediately. In addition, magnetic fields can also operate the two LMs’ coalescence on the water surface (Fig. 6(b)).^120^ To combine LMs in batches quickly, many special devices have been designed; *e.g.*, an automatic coalescence and splitting system (CSS) can achieve the preparation, coalescence and splitting of LMs by gravity and centrifugal force, which can produce optimal parameters in the LMs.^19^ Similarly, a ramp can be used to coalesce two LMs, which is designed with several stages to determine the speed of the LMs.^123^ Castro reported a simple method to produce composite LMs continuously (Fig. 6(c)).^15^ The inclination angle of the slide and the width of the orifice gap could control the coalescence of two LMs. Moreover, the merging of two different types of LMs can also be achieved (Fig. 6(d)).^27^ Acoustic levitation can also drive two LMs to move toward each other, collide and eventually coalesce into a single marble, which can trigger a chemical reaction (Fig. 6(e)).^45^ The pressure gradient on the LM surface formed a liquid bridge, which leads to the merging. The impacting of two LMs can also coalesce two LMs (Fig. 6(f)).^121^

Although a magnetic field could be applied to coalesce LMs, this method is only suitable for the LMs coated with magnetic particles. Thus a DC electric field can be applied to operate the coalescence of nonmagnetic LMs (Fig. 6(g)).^124^ When the DC voltage exceeds the threshold value, the two LMs can coalesce efficiently. The threshold value is significantly influenced by the diameter of the coating particles and the surface tension of the liquid core. If the voltage is large enough, the coalescence of multiple marbles could occur.

### 3.5 The splitting of LMs

**Fig. 7:**
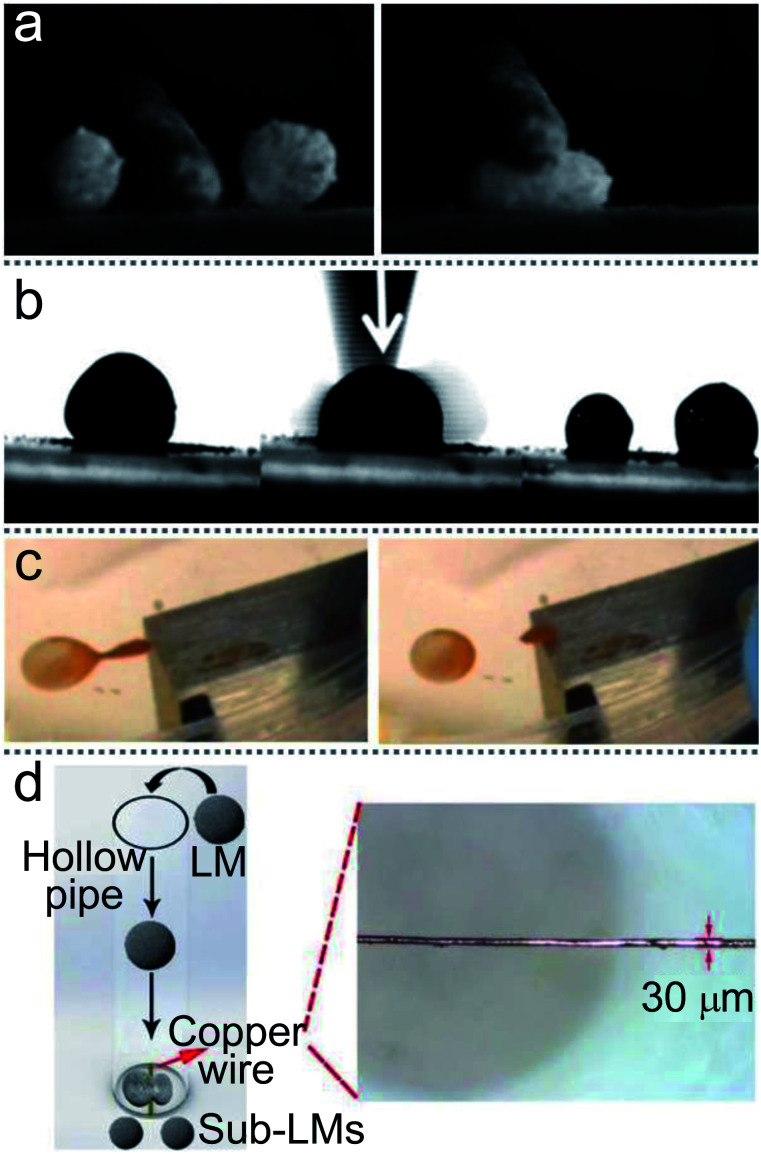
Various methods of separating liquid marbles. (a) The division of marbles with a solid stick. Reproduced from ref. 71 copyright 2006, The Royal Society. (b) A marble is cut using a scalpel blade to form two separate coated droplets. Reproduced from ref. 66 copyright 2012, John Wiley and Sons. (c) The splitting of Janus liquid marbles to form daughter marbles by a magnet bar. Reproduced from ref. 15 copyright 2016, American Chemical Society. (d) The splitting process of LMs by copper wire. Reproduced from ref. 19 copyright 2019, John Wiley and Sons.

LMs’ splitting is a typical process in microfluidic applications. LMs are coated by multiple layers of particles and there are enough particles to coat the newborn LMs, even the total surface area of the LMs increases after slicing. An LM coated with hydrophobic Fe_3_O_4_ nanoparticles can be simply split into two sub-LMs using a spatula.^125^ Moreover, Aussillous cut an LM with a solid stick or even a finger (Fig. 7(a)).^71^ Janus droplets, wrapped with two different powders, have been split into halves using a needle.^27^ Sivan rolled an HCl treated Galinstan droplet on an 80 nm WO_3_ powder bed to form LMs and then cut it using a scalpel blade (Fig. 7(b)).^66^ Furthermore, a magnetic bar can also remotely split a Janus droplet into sub-LMs. If the LM’s shell contains iron oxide, the magnet could split the LM and reduce the marble size, which can also decrease the density of surface particles (Fig. 7(c)).^15^ This technique overcomes the limitations of other splitting processes which could potentially disrupt the surface coverage of the liquid marble. A copper wire with a diameter of 30 μm was tightly straightened in the path of falling LMs to split LMs and may be more valuable in actual production (Fig. 7(d)).^19^ When an LM falls from a certain height and impacts the copper wire through the sphere center, the LM can be cut into two sub-LMs within 10 ms. The surface particles of the sub-LMs would be self-rearranged.

The coalescence and splitting of two or multiple LMs are key factors to achieve extensive applications, such as stoichiometric chemistry and biomedical applications. The coalescence of the LMs can be applied to accurately mix the reactants of two LMs. The splitting process can remove the waste after reactions, especially the splitting of metabolites during the long-time culture of cells.^19^

## 4 Liquid marbles operations

LMs’ shells could prevent the inner liquid from wetting the carrier surface which can be solid or liquid. In other words, LMs can transport the required liquid on solid or liquid surfaces, exhibiting extremely low friction with carrier surfaces. There are many methods to drive LMs: Marangoni propulsions, electric fields, magnetic fields, ultrasonics, *etc*.

### 4.1 The gradient of the carrier liquid

In nature, Marangoni propulsions can enable *Stenus* to march on air–water interfaces. Marangoni effects are mainly due to the gradient on the liquid surface. Thus the transport of LMs also arises from the gradient of the carrier liquid, since different surface tension exists between the front and rear sides of the marble. And a gradient threshold of the surface tension is estimated, which can achieve the marble’s self-propulsion.^131^

**Fig. 8:**
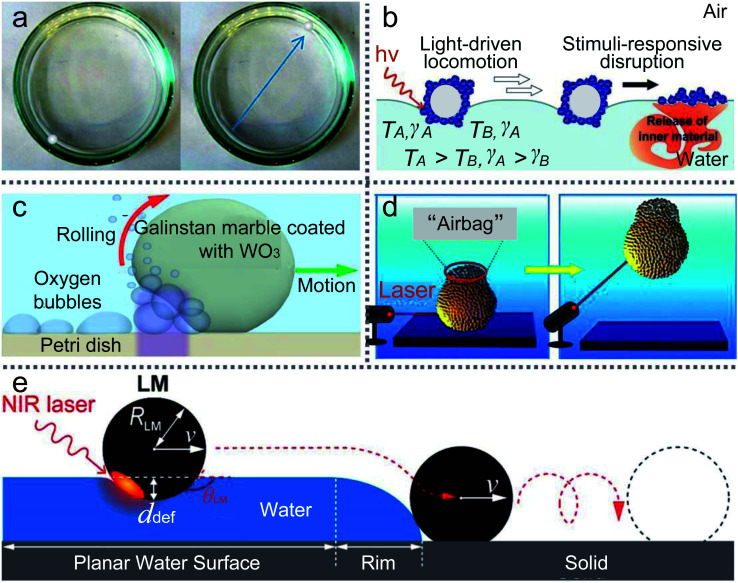
The manipulation of LMs by Marangoni propulsions. (a) Marangoni flows caused by the evaporation of alcohol inside marbles. Reproduced from ref. 126 copyright 2015, American Chemical Society. (b) Light-driven deliveries and release of materials using liquid marbles. Reproduced from ref. 127 copyright 2016, John Wiley and Sons. (c) Photo-chemically induced motion of liquid metal marbles in H_2_O_2_ solution. Reproduced from ref. 128 copyright 2013, AIP Publishing. (d) Remote manipulations of marbles in water by near-infrared laser. Reproduced from ref. 129 copyright 2016, American Chemical Society. (e) Transport of materials from water to solid surfaces using liquid marbles. Reproduced from ref. 130 copyright 2017, American Chemical Society.

A floating marble containing ethanol solution would drive itself since the gradient is caused by the evaporation of the inner liquid (Fig. 8(a)).^126^ The initial velocity of the marble would rise with the increase in the ethanol concentration in the marble. In contrast, when adding ethanol to the carrier liquid, the marble’s velocity would decrease dramatically. The gradient of surface tension can be varied by the ethanol concentration in the carrier liquid.^132^ Adding ethanol to the liquid substrate would reduce surface tension and weaken the motion of LMs. For example, Ooi moved an LM containing a volatile compound by the gradient of surface tension.^133^ The volatile compound evaporates from the marble and is adsorbed by the carrier liquid. The Marangoni flow would appear on the water surface and push the LM. The self-propulsion also occurs when the LM is filled with sulfuric acid.^134^ But the motion of sulfuric acid marbles would be more sensitive to the thermal field than evaporation, which may initiate an exothermic chemical reaction.

Besides the dissolution of chemicals, light can also trigger the gradient of the carrier liquid. In 2016, Paven described an experiment using light to transport LMs on the water surface and release the inner liquid at the destination (Fig. 8(b)).^127^ A NIR laser or the sun can illuminate floating marbles and convert light into heat, which would also cause a gradient of surface tension on the liquid surface. The NIR laser could control the delivery position, direction and velocity of LMs. The LMs’ motion would vary along with the incident angle of the NIR laser. For example, when the incident angle is approximately 45°, LMs would move immediately on the water surface. However, when the incident angle is changed to 90°, the marble may be trapped in one spot. Therefore, the light-driven LM could be applied to push or pull small objects. Kawashima investigated the marble delivery and used NIR light to drive marbles encapsulated with hydrophobic PPy powders.^136^ Excellently, Kavokine deposited marbles containing photosensitive surfactant on the water surface and the marbles were transported back and forth by UV/blue light.^137^

Much literature has reported the Marangoni flow transport of LMs, but the opposite phenomenon is also found to be able to transport LMs: the anti-Marangoni flow, driven by surface deformation.^138^ When marbles float on a thin liquid substrate, whose thickness is close to the capillary length, the anti-Marangoni flow would move the marbles in the opposite direction to the surface tension gradient.

Researchers prefer to study the movement of LMs on a liquid or solid surface, but rarely study the LMs’ movement inside the carrier liquid. UV light could control the LM in a H_2_O_2_ solution (Fig. 8(c)).^128^ The marble is coated with WO_3_ nanoparticles and the UV source is set at one side of the marble, which can generate oxygen bubbles. The oxygen bubbles would push the marble and the marble’s motion is determined by the concentration of H_2_O_2_, the intensity of UV light and the marble dimensions. Similarly, a near-infrared laser could also initiate the movement of marbles in water (Fig. 8(d)).^129^ The marble can be ascended, shuttled, horizontally moved and even suspended in the liquid with the laser’s direction. When focusing the laser’s spot on the marble surface, a black “airbag” would appear on the top of the marble. The “airbag” can produce extra buoyant forces to lift the marble in the liquid. After removing the laser, the marble stops ascending and falls to the solution bottom. More interestingly, if the laser intermittently irradiates the marble, the marble can repeatedly ascend in the water until the “airbag” burst.

More than that, the NIR laser or weak airstream can remotely transport the LMs from the water surface to a solid surface (Fig. 8(e)).^130^ Since the NIR laser’s irradiation could control the LM’s movement on the water surface, the LM can be slipped over the meniscus on the edge of the water surface and is transferred to the PMMA substrate. And the weak airstream could provide the LM with sufficient kinetic energy to climb over the meniscus of the liquid.

### 4.2 Electric fields

Electric fields can deform the shape of marbles and move LMs. Aussillous and co-workers used an electric stick of Teflon to approach an LM and check the effect of electrostatic fields.^71^ They observed a consecutive bouncing motion of the LM and small droplets were ejected. Moreover, a finger electrode structure could control the motion of marbles.^73^ Meanwhile, the contact angle of LMs on the smooth solid surface can be changed by adjusting the AC or DC bias voltage. But when the voltage is increased too high, the marble would burst.^139^

**Fig. 9:**
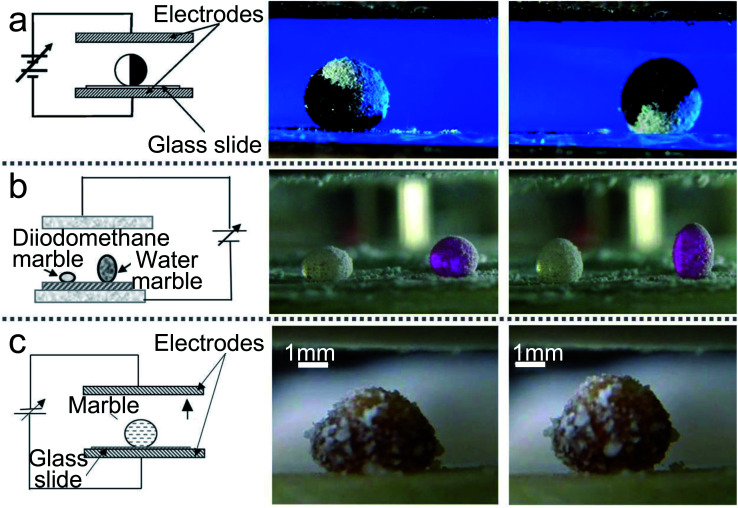
Liquid marbles are actuated by electric fields. (a) Electrical actuation of liquid marbles and the rotation of the marble by the electric field. Reproduced from ref. 27 copyright 2011, American Chemical Society. (b) The behavior of composite marbles in the electric field. Reproduced from ref. 80 copyright 2012, AIP Publishing. (c) The marble’s shape is deformed by electric fields. Reproduced from ref. 135 copyright 2015, Springer Nature.

The charge electrophoretic motion of Janus marble can be induced with AC electric fields.^140^ The Janus marbles’ velocity is determined by the field strength and the salt concentration of the inner liquid. Correspondingly, McHale applied AC electric fields to LMs and the voltage was up to 200 V with the frequency sweep from 1 to 250 Hz.^72^ He found two different results: at low frequencies, the motion of LMs is up and down; as the frequency increased, the motion is transformed into the resonant oscillation. The precise resonance frequency depends on the marble volume. When a Janus LM is manufactured with two different powders, semiconductor and dielectric, the Janus LMs could be activated with electric fields (Fig. 9(a)).^27^ Moreover, the Janus LMs deposited on glass slides can be rotated with the electric field. When the electric field attains the value of *E* ≈ 5 × 10^5^ V m^−1^, the Janus LMs start rotating. If the electric field attains the value of *E* ≈ 7 × 10^5^ V m^−1^, the Janus LMs are destabilized and destroyed (Fig. 9(b)).^80,81^

Electric fields can also induce a surface tension gradient on the liquid surface. When imbalanced forces overcome the friction, the motion of floating the LM would be achieved.^141^ DC electric fields can operate the LM on the silicon oil surface and change its shape from spherical to prolate-spheroid.^142^ If the electric fields are removed, the marble would return to the spherical shape. The electric fields can not only deform the marble containing petroleum (Fig. 9(c)),^135^ but can activate a droplet to climb onto a composite marble. The composite marble is placed on a superhydrophobic surface located between two plain electrodes and the electric field is increased from 0 to 10^6^ V m^−1^. When the electric field reach 7 × 10^5^ V m^−1^, the droplet starts to climb on the composite marble. Increasing the electric field to the value of 10^6^ V m^−1^, the water droplet arrives at the top of the LM.

DC electric fields can stimulate the coalescence of marbles.^124^ The required DC voltage for coalescing LMs depends on the particles and the surface tension of the inner liquid. The electric voltage deforms the liquid interfaces to form a bridge and initiate the LMs’ coalescence. In addition, sufficient voltage can trigger the coalescence of 3, 4 and 5 marbles.

Manipulating LMs is fundamental to various applications. LMs are non-stick droplets presenting extremely low friction on the supporting surface.

### 4.3 Magnetic fields

**Fig. 10:**
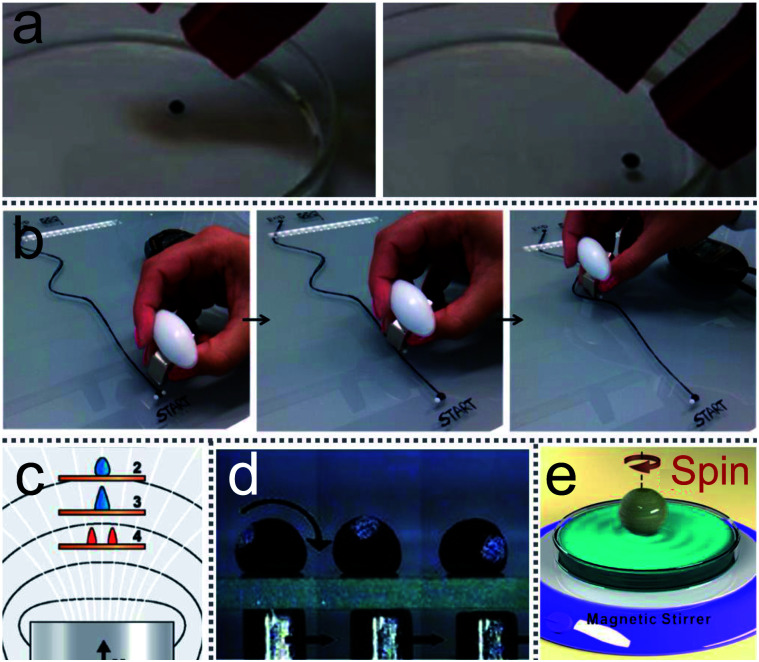
Magnetic field-driven motion of liquid marbles. (a) Magnetically driven LMs on the water surface. Reproduced from ref. 143 copyright 2017, American Chemical Society. (b) The twisting movement of a magnetic liquid marble using a magnetic bar. Reproduced from ref. 105 copyright 2015, American Chemical Society. (c) The marble’s deformation under magnetic fields. Reproduced from ref. 143 copyright 2017, American Chemical Society. (d) Magnetic field-driven motion of a marble on a glass slide. Reproduced from ref. 60 copyright 2010, John Wiley and Sons. (e) A spinning LM on water surface under external rotating magnetic fields. Reproduced from ref. 144 copyright 2016, American Chemical Society.

Although many microfluidic “lab-on-a-chip” devices have been designed to operate liquid in miniaturized chemical processes, there are some published studies to describe another method to manipulate liquid by LMs (Fig. 10(a)–(c)).^105,143,145^ Magnetic LMs are fabricated with a mixture of FD-POSS and Fe_3_O_4_ nanoparticles, which could be used as miniature magnetic reactors (Fig. 10(d)).^60^ The reactors can be moved in different directions and opened/closed repeatedly by magnetic fields. A magnetic bar can transport the magnetic LM on the flat or curved surface, which facilitates the generation of topologically complex microfluidic systems.^61^

Khaw investigated the force on the magnetic LMs by varying the magnetic flux density, flux density gradient, the concentration of magnetic particles and the speed of marbles.^145^ They found the magnetic force and friction are the main factors to control the floating LMs. More interestingly, Han considered the magnetic actuation of LMs only allows a simple mechanical motion, such as linear motion. Thus he changed the trajectory of the magnetic bar from straight to circlular using a commercial stirrer (Fig. 10(e)).^144^ Consequently, the magnetic LMs can be operated in both 2D and 3D under magnetic fields.

### 4.4 Ultrasonics

Ultrasonics can levitate and operate LMs without any contact, and can merge multiple LMs and trigger chemical reactions .^45^ Driven by the ultrasonics, the liquid marbles move toward each other, collide, and eventually coalesce into a larger single marble. In the sound field, the two liquid marbles would approach each other before the surface-protruding structures of the main body contact. The liquid cores of the marbles remain isolated without coalescence even though the two marbles have been in contact with each other. Ultrasonics can cause a surface tension gradient on the LMs’ surfaces, which would form a liquid bridge and result in the LMs’ coalescence. During levitation, the two liquid marbles are bound to each other, and their liquid cores remained isolated for a certain period.

**Fig. 11:**
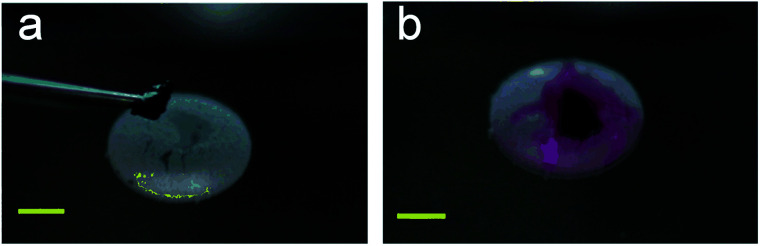
Levitation and operations of marbles by ultrasonics. The on–off motion of the LM’s shell is controlled by acoustic levitation. Reproduced from ref. 59 copyright 2015, American Chemical Society.

Moreover, when increasing the ultrasonic intensity, the shape of marbles would change and the on–off motion of the LM’s shell would be controlled (Fig. 11(a) and (b)).^59^ During the levitation, the gravitational force of the LM is balanced by the acoustic radiation force exerted on the LM’s surface. The liquid marble is being continuously deformed from a quasi-spherical shape to an oblate spheroid with an increase in the sound intensity, since the shape of a LM is determined by the competition between surface tension and acoustic radiation pressure.

Although there are still some problems (see Table 3) in LMs’ manipulation, the new idea of manipulating LMs has been put forward continuously.

**Table 3:** Methods of manipulating LMs

Methods	Advantages	Disadvantages
The gradient of the carrier liquid	Directional operation; remote control; adjustable speed	2D operation; liquid volatilization; inaccurate volume; suitable for use on liquid surfaces or in liquids
Electric fields	Directional operation; remote control; adjustable speed; 3D operation	For solid surfaces; energy consumption
Magnetic fields	Directional operation; remote control; adjustable speed; 3D operation	Valid for magnetic materials only
Ultrasonics	Remote control; adjustable speed	1D operation; imprecise control

## 5 Applications

### 5.1 Miniature reactors

**Fig. 12:**
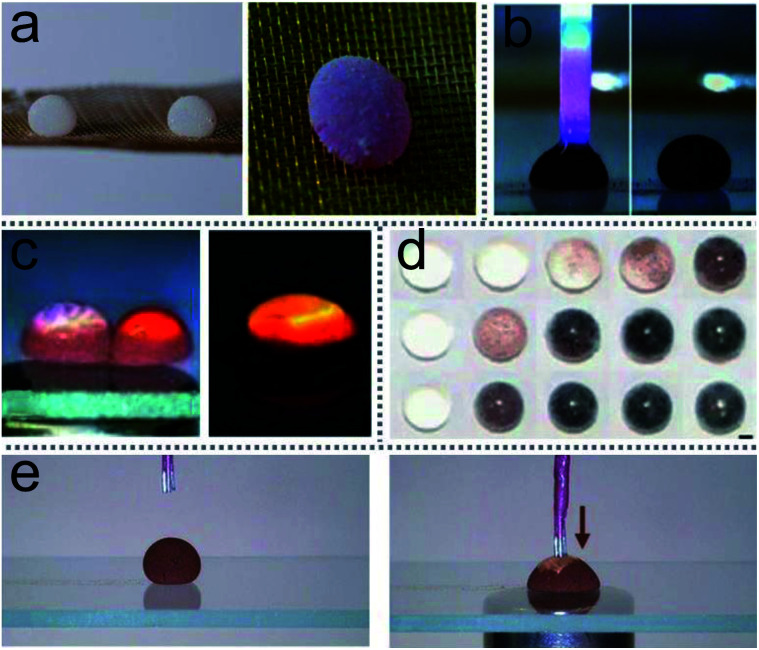
Liquid marbles as microreactors. (a) The microreaction of phenolphthalein and NaOH solution in liquid marble triggered by acoustic levitation. Reproduced from ref. 45 copyright 2017, American Chemical Society. (b) Chromatographic analysis of liquid in an opened liquid marble. Reproduced from ref. 60 copyright 2010, John Wiley and Sons. (c) A chemiluminescence reaction occurs by coalescing two liquid marbles which contain different reagents. Reproduced from ref. 60 copyright 2010, John Wiley and Sons. (d) Liquid marbles as microreactors for the silver mirror reaction. Reproduced from ref. 44 copyright 2015, John Wiley and Sons. (e) Electrochemical detections. Reproduced from ref. 28 copyright 2014, John Wiley and Sons.

LMs as miniature reactors have many advantages, such as reducing chemical reagents, precisely controlling reaction conditions and accelerating reaction rates. More importantly, LMs could be extended to trigger reactions with multiple reagents (Fig. 12(a)), such as liquid chromatographic analysis (Fig. 12(b)) and chemiluminescence reactions (Fig. 12(c)).^60^ Magnetic manipulations enabled the inner liquid of marbles to communicate with the outside environment. For example, a magnetic bar can open the LM’s shell to allow the extraction and addition of the inner liquid for further analyses. On-line electrochemical detection has been widely used in many microfluidic systems. The detection can also be performed in an opened LM, which uses an Ag/AgCl wire and two platinum wires as the three-microelectrode probe to monitor the reaction in the LMs (Fig. 12(e)).^28^ Thus LMs can be applied as a surface-enhanced Raman scattering (SERS) platform.^46^

LMs as miniature reactors can accelerate the reaction rate since the diffusion of reactants is enhanced by droplet oscillation during LMs’ coalescence. Acoustic levitation can induce micro-reactions in the LM.^45^ Two LMs held by the levitator contain 10 μL phenolphthalein and NaOH solution, respectively. Once the levitator works, the two marbles would coalesce into a larger marble and the inner liquid rapidly turns pink, which indicates that NaOH solution has mixed with the phenolphthalein. The shell of silica-particle-based LMs provides reaction substrate surfaces to conduct the silver mirror reaction (Fig. 12(d)).^44^ When the silver mirror reaction takes place in the miniature reactors, the color of the LMs changes rapidly.

Another advantage of LMs as miniature reactors is that the reaction rate can be adjusted. The reaction rate is mainly determined by the solution concentration, activation energy and temperature. The microreactor can be heated using an irradiation laser when the microreactor is fabricated with graphene powders and has photothermal properties.^26^ Through increasing the laser power, the microreactor’s surface temperature could reach 135 °C and its inner liquid’s temperature can reach 74 °C.

### 5.2 Biological incubators

**Fig. 13:**
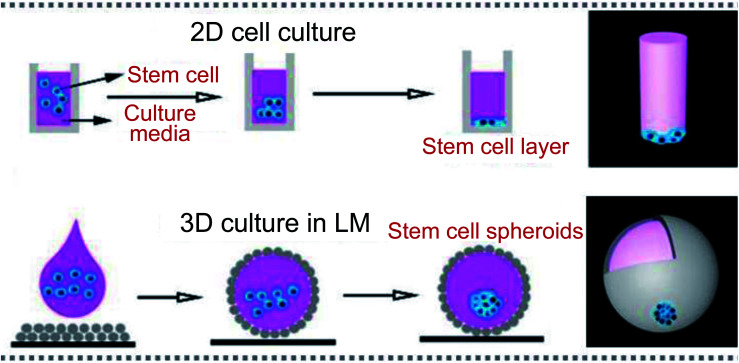
Liquid marbles as biological incubators. Culturing cells in a 2D format and 3D incubator. Reproduced from ref. 19 copyright 2019, John Wiley and Sons.

**Fig. 14:**
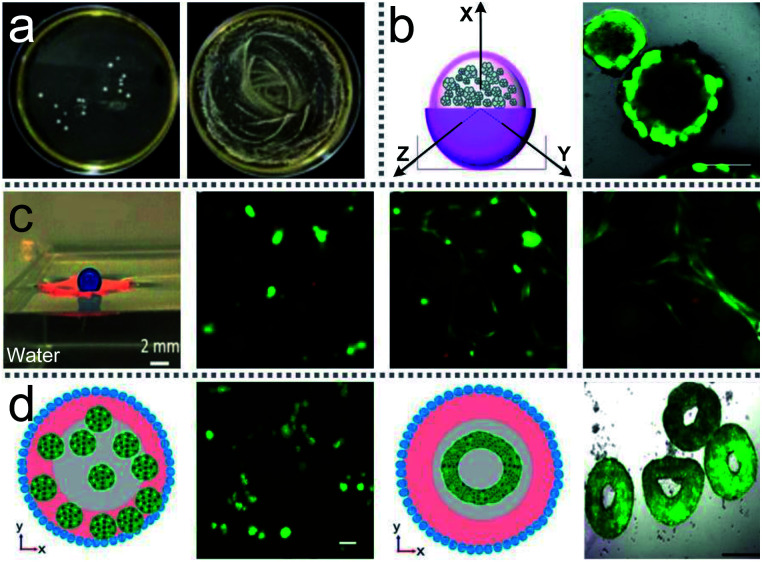
Liquid marbles as biological incubators. (a) *S. cerevisiae* culture from a liquid marble. Reproduced from ref. 2 copyright 2013, Elsevier. (b) Cancer cell spheroid formation in a liquid marble. Reproduced from ref. 48 copyright 2012, John Wiley and Sons. (c) The GelMA structure on water surface. Reproduced from ref. 50 copyright 2016, American Chemical Society. (d) Physical properties of liquid marbles for the generation of spheroids and toroids. Reproduced from ref. 53 copyright 2017, Springer Nature.

Due to the natural advantages of the LM structure, such as preventing the liquid core from contacting with outside surfaces, allowing gases to pass freely through the shell, the marbles can be used as biological incubators. The porous shell allows gases to enter and exit from the LMs freely, just as LMs can breathe, which provides the basic living conditions for microorganisms. For example, Wang constructed LM-based 3D stem cell spheroids, which can provide approximately 3-fold cell viability compared with conventional spheroids (Fig. 13).^19^ Tian selected two types of microorganisms, which have different responses to oxygen, to conduct biological culture. The LM as a respirable incubator provides a more suitable environment for cells to grow than that in bottles: cell concentrations increase more rapidly in LMs (Fig. 14(a)).^2^ The cancer cells could be cultured in LMs, which opened new avenues in cancer research (Fig. 14(b)).^48^ There exist many advantages with LMs as cancer cell spheroids (CCSs): quick cell aggregate formation, simple operation, cost-effective, *etc*. This method can also be extended to culture other cells. For example, Li used LMs to culture tumors (lung cancer stem cells).^3^ Sarvi conducted another biological application of LMs: culturing of embryoid bodies (EBs) from embryonic stem cells (ES cells).^51^ Furthermore, polytetrafluoroethylene LMs can provide a suitable microenvironment to culture murine embryonic stem cells.^49^

To exploit LMs as biological incubators, factors such as particle size, liquid marble volume and cell density have been investigated. Research shows that smaller particles generally can produce more stable biological incubators. The greater cell density and size of LMs can reproduce more cells. Moreover, the volume of the biological incubator can also be flexibly adjusted by coalescing two (or more) LMs.^2^ Ledda compared the maturation of sheep oocytes cultured in LMs and four-well Petri dishes, respectively.^52^ These sheep oocytes showed similar expansion but LMs would reduce the reagent consumption.

LMs can float on the water, which would extend the biological incubator’s lifetime and achieve nutrient exchange with the surrounding aqueous medium (Fig. 14(c)).^50^ Floating LMs can culture olfactory ensheathing cells (OECs). Moreover, co-culturing OECs with Schwann cells and astrocytes in the floating LMs can form complex cell structures.^31^ Another method to extend the lifetime of biological incubators based on LMs has been proposed by Vadivelu, which breaks the time limit on the culturing process (Fig. 14(d)).^53^ A hydrogel sphere is put in the LM which served as storage for the nutrition solution. The hydrogel sphere can release nutrients into the culture medium for inner cells.

To accelerate the growth rate of cells, graphene LMs as photothermal biological incubators can achieve precise temperature control by tuning laser power.^26^ This method could guarantee the marble’s surface temperature between 21–135 °C and the inner liquid temperature between 21–74 °C, which achieves a 12-fold superior culture rate than that at room temperature.

Marbles coated with traditional powders such as graphene and carbon black hardly allow visible light to pass through and reach the inside of LMs. Compared with traditional marbles, a biological incubator coated with magnetic lanthanide-doped upconversion nanoparticles (UCNPs) can be used for photodynamic therapy and convert near-infrared light into visible light, which can accelerate drug screening and culture cancer cells.^54^ The marbles allow the light to pass through and can carry out photon-induced reactions to generate nutrients to promote cell viability.

### 5.3 Unloading liquid

**Fig. 15:**
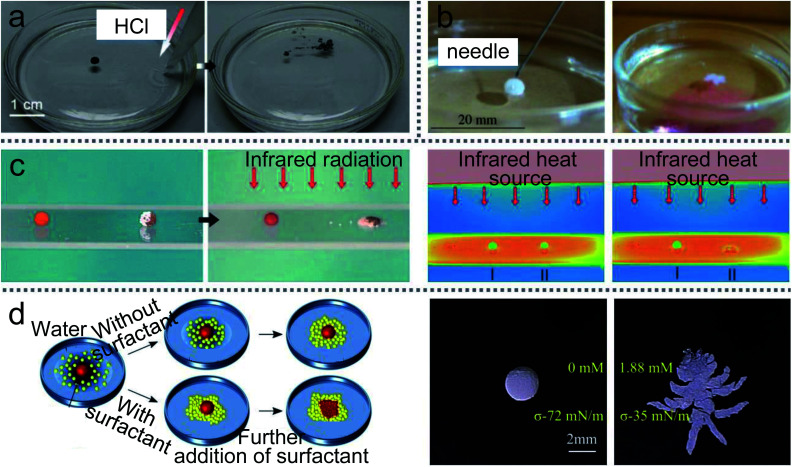
Unloading the inner liquid of marbles. (a) The rupture of liquid marbles on the water surface after the addition of HCl solution. Reproduced from ref. 99 copyright 2012, John Wiley and Sons. (b) A marble is punctured by a needle. Reproduced from ref. 20 copyright 2009, John Wiley and Sons. (c) Thermo-triggered rupture of a Janus liquid marble. Reproduced from ref. 146 copyright 2014, John Wiley and Sons. (d) The rupture by the different surface tension of the liquid substrate. Reproduced from ref. 132 copyright 2019, American Chemical Society.

LMs can be remotely controlled to unload the inner liquid when reaching the destination, which can initiate chemical reactions with the carrier liquid (Fig. 15(a)).^99^ For example, LMs containing one reactant floated on the surface of another reactant.^147^ When the LM disintegrates under the action of magnetic fields, the two reactants can mix and start the chemical reaction. A needle can also be used to pierce the LMs on the water surface to unload the internal reagents and conduct chemical reactions (Fig. 15(b)).^20^ When a Janus LM is composed of a droplet, a magnetic semi-shell (brown) and a nonmagnetic semi-shell (white), it can be controlled by a magnetic bar. The marble can rupture upon IR irradiation when the temperature-responsive magnetic semi-shell contacts with the glass substrate (Fig. 15(c)).^146^ When the LM, which is coated with novel core/shell-structured responsive magnetic particles, is exposed to UV light, it would remotely trigger a rupture and the inner liquid would leak.^85^ Similarly, centrifugal force and near-infrared (NIR) light can also induce LMs to release liquid at the destination.^19,148,149^

When the marble is prepared by 1-bromo-3-fluoro-4-iodobenzene (BFI) powder with a melting point of 46.5 °C, the LM can float on the surface of carrier water at room temperature.^150^ When the temperature of the carrier water is above the melting point of the BFI powder, the LM disintegrated. Comparatively, a cooling method can also break the LMs.^29^ When thermo-responsive LMs coated with PNIPAM powder are transferred to the water surface, they can remain stable on the water surface for more than one day at room temperature. But when the water is cooled, the LMs disintegrate since the wettability of the PNIPAM powder increases. Reducing the surface tension of water by adding surfactant can lead the LMs to disintegrate (Fig. 15(d)).^132^ The required surface tension and surfactant concentrations of the water surface are determined by the volume of the floating LMs.

### 5.4 pH sensors

LMs are highly sensitive to external acid–base stimuli. Researchers attempt to apply them as pH sensors to detect the environment. For example, an LM fabricated with HFUA powder could be used as a pH sensor for the carrier liquid.^147^ The LM could steadily float on the surface of a neutral or acidic solution, but it would disintegrate once NaOH solution is added to the carrier liquid. Additionally, when the LMs are prepared with micrometer-sized silica particles (PAaH-SiO_2_), the LM surface’s hydrophobicity/hydrophilicity can be changed by external acid–base stimuli.^93^

**Fig. 16:**
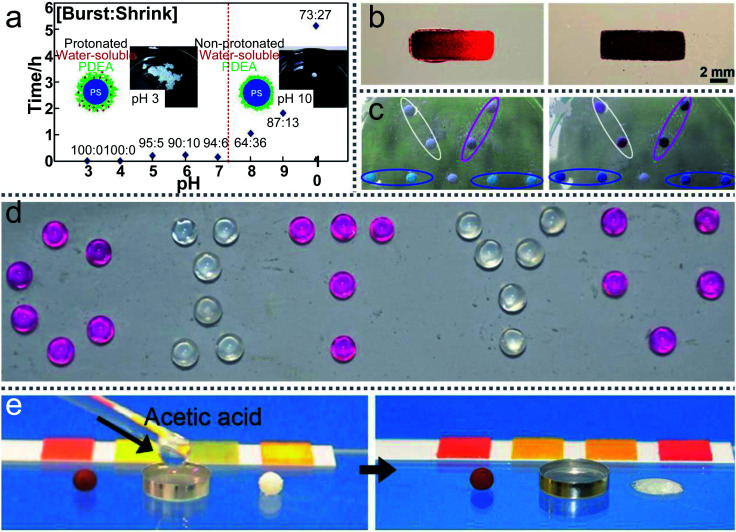
Liquid marbles as pH sensors. (a) The average lifetimes of liquid marbles on the surface of liquids with various pH values. Reproduced from ref. 151 copyright 2011, American Chemical Society. (b) A floating pH meter. Reproduced from ref. 50 copyright 2016, American Chemical Society. (c) Gas detections and reactions. Reproduced from ref. 57 copyright 2010, Elsevier. (d) The liquid marbles with phenolphthalein solution (“C”, “T”, “U”) are exposed to ammonia gas. Reproduced from ref. 3 copyright 2017, John Wiley and Sons. (e) Gas-triggered rupture of a liquid marble. Reproduced from ref. 146 copyright 2014, John Wiley and Sons.

Furthermore, the LMs coated with PDEA-PS latex powder can exhibit long-term stability on a liquid surface whose pH is above 8 (Fig. 16(a)).^151^ However, when the LM is transferred onto the surface of an acidic liquid, it would disintegrate within 10 min due to the dispersal of the PDEA-PS latex particles. A novel floating pH meter coated with hydrogels could free-float on the liquid surface (Fig. 16(b)).^50^ When transporting the pH meter to the liquid surface and increasing the pH of the surrounding liquid from 5 to 9, the color of the floating pH meter changes from vivid red to dark brown. Through comparing the pH of the liquid with the LMs’ color, the pH of the liquid can be monitored in real-time.

The marbles could also detect the surrounding gas types and contents, such as NH_3_, HCl, and formaldehyde vapor.^3,57,68,146,152,153^ The porous shell enables the gas to dissolve into the LMs (Fig. 16(c)).^57^ For example, when an LM loaded with phenolphthalein indicators is placed on the ammonia solution surface, the LM would become pink since ammonia gas penetrated through the LM’s shell (Fig. 16(d)).^3^ There also existed other indicators to detect gas types such as a metal salts solution of CoCl_2_ and CuCl_2_. When the ammonia gas dissolves into the metal salts solution, a sustained color change would appear in the LMs. In addition, a fluorescent pH indicator can be loaded in the marble for HCl gas sensing. When the LM is exposed to HCl vapor and illuminated by UV light, the fluorescent pH indicator would show a clear color change. Similarly, a marble loaded with a mixture of ammonia acetate, acetic acid and acetylacetone can be used to detect formaldehyde vapor.^153^

Multi-responsive LMs coated with a magnetic semi-shell and a nonmagnetic semi-shell have been newly developed. The Janus LMs would rupture once exposed to ammonium hydroxide or acetic acid vapor, thus the marble could be used as a sensor to visually detect acidic/basic vapors (Fig. 16(e)).^146^ Moreover, the LMs have potential as practical gas emission indicators in the workplace, such as monitoring ammonia and amine emission in the printing industry.

### 5.5 Other applications

**Fig. 17:**
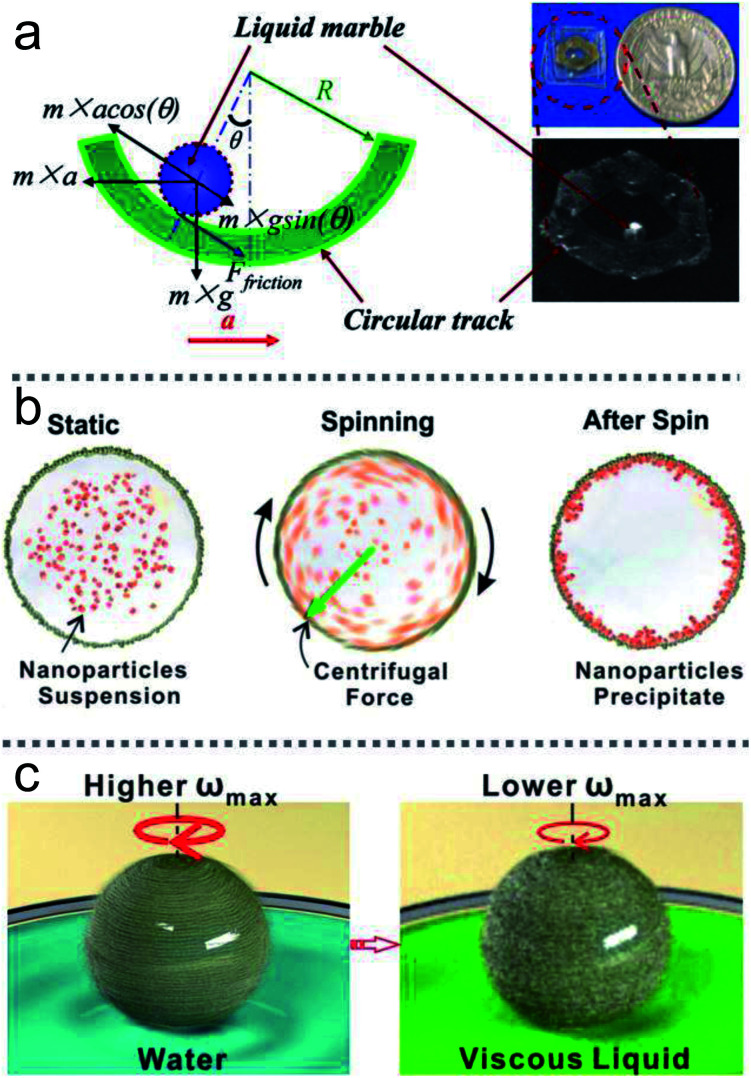
Liquid marbles as precision instruments. (a) Accelerometers using liquid marbles. Reproduced from ref. 94 copyright 2010, AIP Publishing. (b) The spinning LM as a micro-centrifuge. Reproduced from ref. 144 copyright 2016, American Chemical Society. (c) The spinning LM as a miniature viscometer. Reproduced from ref. 144 copyright 2016, American Chemical Society.

LMs are endowed with special functions for unique properties and can be used as micro-precision instruments, such as gas sensors, accelerometers, pressure sensors, *etc*. For example, LMs can be used as a low-frequency accelerometer to detect an object’s motion (Fig. 17(a)).^94^ The LM would reside at the bottom of the channel without an external acceleration. Once an external force appears, the angular displacement of the LM represents the direction and magnitude of the external acceleration.

Apart from accelerometers, LMs can be utilized as microcentrifuges or microviscometers (Fig. 17(b) and (c)).^144^ When a rotating magnetic field drives LMs coated with Fe_3_O_4_ nanoparticles, the LMs would have rotational motion, and could be used as microcentrifuges and microviscometers. The relative liquid viscosity can be judged by the spinning speed of the marble. LMs floating on the water surface could immediately reveal the presence of contaminants, such as oils and petroleum, since contaminants can decrease the surface tension of the carrier liquid.^11^ Similarly, the method can be used for a heavy metal ion sensor when the LMs are coated with WO_3_ nanoparticles.^66^

**Fig. 18:**
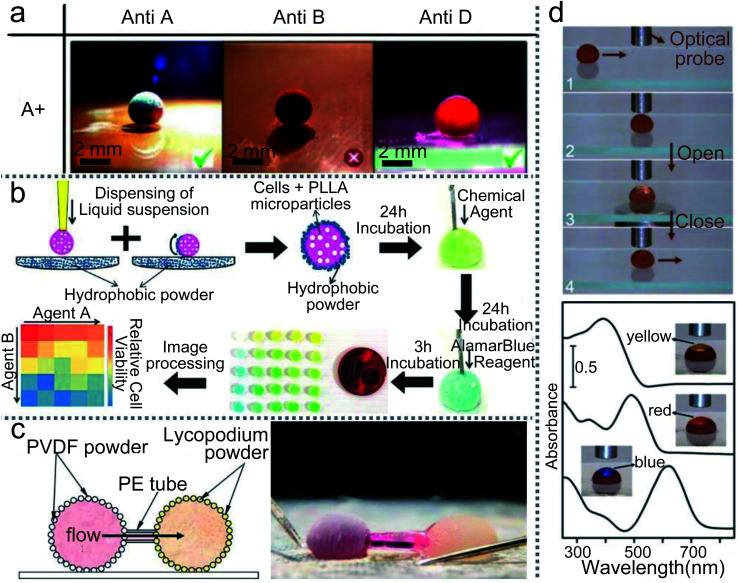
Liquid marbles as precision instruments. (a) Rapid blood typing. Reproduced from ref. 69 copyright 2011, John Wiley and Sons. (b) High-throughput drug screening. Reproduced from ref. 154 copyright 2014, John Wiley and Sons. (c) Micropump based on liquid marbles. Reproduced from ref. 88 copyright 2010, AIP Publishing. (d) Optical detections. Reproduced from ref. 100 copyright 2012, Springer Nature.

LMs have more applications in biological detection, such as rapid blood typing, drug sensitivity tests and drug screening. Three “blood marbles” are prepared with blood samples and a hydrophobic powder: precipitated calcium carbonate (PCC). Three antibody solutions (anti-A, anti-B and anti-D) are injected into the three blood marbles to initiate the hemagglutination test (Fig. 18(a)).^69^ Once the hemagglutination reaction occurs, the initial uniform red color of the blood marbles would separate into two discernible parts: light-red and dark-red colors. These colors indicate the corresponding antigen on the surface of red blood cells (RBCs). At present, drug sensitivity tests are usually performed in a monolayer culture system which has limited value in predicting the clinical efficacy of chemotherapeutic drugs.^3^ LMs could be utilized as 3D cell culture systems for cells rather than monolayers. Furthermore, when the LMs are encapsulated with PLLA microparticles, they can screen the drugs on anchorage-dependent cells (Fig. 18(b)).^154^

The magnetic LM could also be applied as a sample carrier in optical detection. When a magnetic bar approaches the LM from below, the top of the LM can be partially opened and then the optical probe can detect the inner liquid in reflection mode.^28^ More interestingly, when the LM is fully opened, the transmission-mode detection of the inner liquid can be performed. The path of the light can be adjusted through flattening the droplet with two hydrophobic glass slides. Thus the light can pass through the droplet and reach the read probe to finish the detection (Fig. 18(d)).^100^

A micropump can be made by using a capillary tube to connect two LMs which are coated with different powders (Fig. 18(c)).^88^ The driving force of the micropump comes from the unbalanced Laplace pressure of the two LMs. When the PVDF-coated and lycopodium-coated marbles are connected by a capillary tube, the pressure gradient of the two marbles would push the liquid from the PVDF-coated marble to the lycopodium-coated marble.

Moreover, LMs can be implemented as signal carriers in collision-based unconventional computing circuits.^34,112^ Boolean values of the inputs are given by the absence (FALSE) or presence (TRUE) of LMs. The LMs can be diverted along different paths to conserve several signals.^112^ The marbles as signal carriers have some advantages such as computing circuits without electronic and mechanical parts. For example, the logic gates can be constructed by observing the marble directly after collision-based computation.^34^

## 6 Conclusions

LMs have attracted the extensive attention of researchers in the past 20 years and achieved fruitful research results, due to their simple preparation process, rich raw materials, unique properties and broad application. In microfluidic applications, LMs can resolve the physical defect of microdroplets: unable to transfer on a solid surface. A series of preparation methods were proposed based on traditional preparation techniques, such as electrostatic formation, rotary centrifugation, *etc*. Many unique properties of LMs have been discovered, especially maneuverability and elasticity, which are beneficial to the application of LMs.

Researchers have made remarkable progress in the application of LMs, such as sensors, miniature reactors, physical/chemical detection, micro-precision instruments and logic gates. In addition, more attention has been paid to LMs’ application in biological incubators, since LMs can provide a microreactor environment. The gas-permeable shell allows the addition and extraction of reactants and products. Although recent years have seen rapid development in LMs research, there still exist many challenges:

(1) There is still a lack of novel manufacturing techniques to ensure the uniformity of LMs volume and composite functional surfaces.

(2) The evaporation of liquid in LMs needs to be further slowed down or even completely restrained, which can ensure the lifetime of LMs and the stability of the solution concentration in LMs. For example, a constant concentration of the nutrient solution is the basic guarantee for microorganisms in the culture process. When the LMs are used as sensors, the concentration of internal indicators reflects the sensitivity of the sensors. In contrast, the evaporation loss or concentration change of reactants in micro-reactions would provide biased reaction results.

(3) To satisfy various applications of LMs, the mechanical stability of LMs in different environments needs to be strengthened.

These challenges should be addressed in the following ways:

(1) The preparation methods of electrostatic formation, self-assembly on water and rotary centrifugation can ensure accurate LMs volume. But these methods cannot ensure the uniformity of the shell. We can add magnetic nanoparticles in the hydrophobic particles to prepare marbles, which can achieve the precise spatiotemporal actuation of microdroplets. When a rotating magnetic field drives the marble, the uneven areas on the marble surface would disappear during rotation.

(2) Many methods have been proposed to decelerate the volatilization rate of LMs. For example, LMs working on the liquid substrate, changing the relative humidity and temperature of surroundings or LMs coated with airtight and dense membrane materials. So far, LMs working in low atmospheric pressure or vacuum, which may give the marbles a longer lifetime, has not been studied.

(3) To make the LM shell more stable, we can use hydrophobic particles of smaller sizes to coat LMs. Powders of smaller sizes are more easily aggregated to form integral polymer films, which can increase its stability. Moreover, we can also mix two different sizes of particles to prepare LMs. Different sizes of powders can fill the gaps and give more support to the LMs’ shells.

Current challenges also point out the future direction of LMs. In the first place, more preparation methods need to be proposed. For example, smaller or larger LMs can be prepared by splitting or merging. Therefore, the density of the LM surface particles can be adjusted more easily. Also, there are few studies about the LMs as signal carriers in collision-based unconventional computing circuits. The ideas of collision-based computing existing from the nineteenth century have been put in an automata framework. Thirdly, LMs can be used to transport powders or liquid on water or solid surfaces, which can be driven by external stimuli such as light, magnetic fields, and electric fields. To control LMs accurately, frictional force in the process of movement cannot be ignored. In many previous studies, LMs can trigger micro-reactions by injecting another liquid in an overall micro-reaction environment. We can experiment with two LMs wrapping different agents and merge them with an external force. The accomplishment of the experiment inspired the creation of Daniell cells, which just need a tiny amount of the electrolyte to light up an LED lamp.

Overall, the LM is a novel medium to break the limit of hydrophobicity. It could guarantee extremely low friction when rolling on solid substrates since the non-stick droplet is coated with micro- or nano-scale particles. Apart from manipulation and transportation, the LMs could also be potentially used as a miniature lab. It is reasonable to believe that LMs with their excellent performance and applications will play a great role in science and technology.

## Author contributions

Yukai Sun and Yelong Zheng conceived the study and collected the literature. All authors were involved in writing and revising the manuscript.

## Conflicts of interest

There are no conflicts to declare.

## Supplementary Material
